# A Study on the Induction of Multi-Type Immune Responses in Mice via an mRNA Vaccine Based on Hemagglutinin and Neuraminidase Antigen

**DOI:** 10.3390/vaccines13010091

**Published:** 2025-01-19

**Authors:** Mengyuan Liu, Yixuan Liu, Shaohui Song, Qiurong Qiao, Jing Liu, Yun Xie, Jian Zhou, Guoyang Liao

**Affiliations:** 1 Institute of Medical Biology, Chinese Academy of Medical Sciences and Peking Union Medical College, Kunming 650118, China; liumengyuan@student.pumc.edu.cn (M.L.); yxliu@student.pumc.edu.cn (Y.L.); shaohuisong@126.com (S.S.); lj_mirror@126.com (J.L.); xieyun91@foxmail.com (Y.X.); 2 Kunming Medical University, Kunming 650500, China; qqrong1998@126.com

**Keywords:** influenza A virus, mRNA vaccine, hemagglutinin, neuraminidase, Th1, Th2

## Abstract

Background: The Influenza A virus (IAV), a pathogen affecting the respiratory system, represents a major risk to public health worldwide. Immunization remains the foremost strategy to control the transmission of IAV. The virus has two primary antigens: hemagglutinin (HA) and neuraminidase (NA). Our previous studies have demonstrated that an IAV NA mRNA vaccine can induce Th1-type immune responses in mice. This research examined the immune responses elicited by an mRNA vaccine targeting both HA and NA antigens in murine models. Methods: In this study, we used two dual-antigen immunization strategies: single-site immunization with an IAV HA+NA mRNA vaccine and multi-site immunization with an IAV HA mRNA vaccine and IAV NA mRNA vaccine. Hemagglutination-inhibiting antibody titer and neutralizing antibody titer in the sera of immunized mice were evaluated, and a viral challenge experiment was conducted. Additionally, the immune responses elicited by the two immunization strategies were characterized using flow cytometry and ELISA. Comparative analyses were performed with mice immunized individually with the IAV HA mRNA vaccine, IAV NA mRNA vaccine, and inactivated vaccine. Results: The results showed that by using a multi-site immunization strategy, mice were able to generate higher levels of hemagglutination-inhibiting and neutralizing antibodies, and were protected in a viral challenge experiment. Moreover, the multi-site regimen also promoted the generation of cytotoxic T cells and maintained a balanced Th1/Th2 immune response. Conclusions: Using mRNA vaccine based on a HA and NA antigen with multi-site immunization strategy can induce higher levels of hemagglutination-inhibiting and neutralizing antibodies, and multi-type immune responses in mice, providing new theoretical and experimental support for advancing upcoming influenza vaccines.

## 1. Introduction

Currently available influenza vaccines include recombinant vaccines, live attenuated influenza vaccines, and inactivated influenza vaccines [1]. These vaccines are produced using various platforms such as egg-based production, cell-based production, and insect cell expression systems. Among them, egg-based production, as the dominant method for influenza vaccine production, faces numerous challenges. It involves a long production cycle and requires a large quantity of qualified chicken embryos [2]. Additionally, during the culturing of the influenza virus, the hemagglutinin (HA) antigen may undergo adaptive mutations, which can reduce vaccine efficacy [3,4]. This method is also unsuitable for individuals with egg allergies [5,6]. The emergence of cell-based production methods has shortened production cycles and eliminated the risks associated with egg allergies, but it still cannot overcome the issue of adaptive mutations in the HA antigen. Studies have shown that IAV H3N2 and IBV strains can undergo mutations during culture [7,8]. Recombinant vaccines produced using insect cell lines to express influenza antigens offer a promising alternative. This method eliminates the need for additional inactivation steps, saving production time, and avoids the issue of antigenic drift. It is also suitable for individuals with egg allergies [9]. However, recombinant vaccines exhibit lower immunogenicity and lack efficacy in children, making them only applicable for adult use [10]. Therefore, there is a pressing need for a more convenient and effective platform to produce influenza vaccines.

mRNA vaccines are widely recognized for their pivotal contribution to combating the COVID-19 pandemic, and in recent years, mRNA vaccines targeting RSV have also been successfully approved [11,12]. This suggests that mRNA vaccines hold promise as an effective preventive measure against influenza, another respiratory virus. Compared to traditional influenza vaccines, mRNA vaccines avoid the issue of antigenic drift in influenza viruses, adhere to a simplified and highly reproducible manufacturing process, and can produce protein complexes that are difficult to generate with other production methods, thus, broadening the range of protection offered by the vaccine [13]. Currently, eight mRNA influenza vaccines have entered clinical trials, and notably, these include not only mRNA vaccines targeting HA but also those targeting NA [14,15,16].

Compared to HA, NA is the second most abundant surface glycoprotein with a lower mutation rate [17,18]. Multiple HA subtypes share the same NA subtype, suggesting that the protection provided by NA antibodies may be broader than that of HA antibodies [19,20]. Furthermore, the immune responses induced by HA and NA differ. HA-specific immunity can clear viral infections, while NA-specific immunity inhibits the release of influenza virus, significantly reducing morbidity and shortening the duration of illness [14]. These findings indicate that inducing immune responses targeting both HA and NA may be key to achieving optimal protection with influenza vaccines [17].

We have previously confirmed that the IAV NA mRNA vaccine can induce Th1-type immune responses in mice [21]. Therefore, in this study, we employed two dual-antigen immunization strategies. One approach followed the previously established design, where the HA and NA antigens were linked using P2A and formulated into an IAV HA+NA mRNA vaccine. Under the second method, a multi-site approach was adopted, injecting the IAV HA mRNA vaccine into the left hind limb and the IAV NA mRNA vaccine into the right hind limb. The results demonstrated that the multi-site immunization strategy induced higher levels of hemagglutination-inhibiting antibodies (HIAb) and neutralizing antibodies (nAb), promoted the generation of cytotoxic T cells, and maintained a balanced Th1/Th2 immune response, providing more comprehensive protection for the mice. This study’s findings deliver new theoretical insights and laboratory support essential for the progression of future influenza vaccine development.

## 2. Materials and Methods

### 2.1. Virus and Cells

The influenza A virus (IAV) strains used in this study were A/Michigan/45/2015 (H1N1) and A/Victoria/4897/2022 (H1N1), which were propagated and purified in chicken embryos and stored at −80 °C until use. The infectious dose for A/Michigan/45/2015 (H1N1) was 10^−6^ for the median cell culture infection dose (TCID_50_) and 10^−3^ for the median lethal dose (LD_50_). For A/Victoria/4897/2022 (H1N1), the TCID_50_ was 10^−4.667^, and the LD_50_ was 10^−4.6^.

The cell line used was MDCK cells, cultured in DMEM (Servicebio, Wuhan, China) supplemented with 10% FBS (Gibco, New York, NY, USA) and 1% penicillin-streptomycin (Gibco, New York, NY, USA).

### 2.2. mRNA Production

The sequences of A/Michigan/45/2015 (H1N1)-HA, A/Michigan/45/2015 (H1N1)-NA, and A/Michigan/45/2015 (H1N1)-HA+P2A+NA were codon-optimized, synthesized (GenScript, Nanjing, China), and cloned into mRNA production plasmids. The plasmids were amplified using *E. coli* Stabl3 (ThermoFisher Scientific, Waltham, MA, USA), and after plasmid extraction, the linearized templates were generated via digestion with BspQI restriction enzyme and purified using DNA magnetic beads (Vazyme, Nanjing, China). Subsequently, in vitro transcription (IVT) was carried out employing T7 RNA polymerase (Vazyme, Nanjing, China), CleanCap (Syngenbio, Nanjing, China), and dNTPs (substituting m1ψ-5′ triphosphate for UTP, Syngenbio, Nanjing, China) to synthesize Influenza A HA mRNA, Influenza A NA mRNA, and a combined HA+NA mRNA for Influenza A. mRNAs were purified using RNA magnetic beads (Vazyme, Nanjing, China) after IVT reaction.

### 2.3. LNP Encapsulation

The prepared mRNA solutions were encapsulated in lipid nanoparticles (LNPs). The mRNA solutions were prepared at a concentration of 200 μg/mL in a 25 mM acetate-acetate sodium (pH 5.5) buffer. The LNP formulation consisted of four components: cationic lipid, phosphatidylcholine, cholesterol, and polyethylene glycol (PEG)-lipid, in a molar ratio of 50:20:38.5:1.5, dissolved in anhydrous ethanol. The mRNA and LNP components were mixed in a 3:1 flow ratio using a microfluidic device, forming an mRNA-LNP mixture. Subsequently, the ethanol was replaced with a 25 mM Tris-HCl (pH 7.5) buffer using a 100 kDa ultrafiltration centrifugal filter, and the solution was concentrated to yield the mRNA-LNP vaccine. The encapsulation efficiency was determined using the Quant-iT^TM^ RiboGreen RNA Kit (ThermoFisher Scientific, Waltham, MA, USA), while particle size and polydispersity were measured with a Nanoparticle Size and Zeta Potential Analyzer (Malvern Panalytical, Malvern, UK). The results showed that the mRNA-LNP vaccine had an encapsulation efficiency of >95%, an average particle size of 90 nm, and a polydispersity index (PDI) of <0.2.

### 2.4. Mouse Immunization

This study employed female Balb/c mice that were 6 to 8 weeks old and classified as specific-pathogen-free (SPF), provided by the Laboratory Animal Center of the Institute of Biophysics, Chinese Academy of Medical Sciences. Six groups were formed by randomly assigning the mice, with *n* = 5 per group. In the HA group, the mice were intramuscularly injected with 30 μg of IAV HA mRNA vaccine in the hind limb; in the NA group, the mice received 30 μg of IAV NA mRNA vaccine; in the HA+NA group, the mice were injected with 30 μg of IAV HA+NA mRNA vaccine in the hind limb; in the HA/NA group, the mice received 15 μg of IAV HA mRNA vaccine in the left hind limb and 15 μg of IAV NA mRNA vaccine in the right hind limb; in the quadrivalent inactivated vaccine group, the mice were injected with a single dose of A/Michigan/45/2015 (H1N1) quadrivalent inactivated vaccine (generated in our laboratory) in the hind limb; and in the LNP group, the mice were injected with an equivalent volume of LNP. The primary immunization was performed on day 0, the booster dose was administered on day 14, and on day 21, after anesthesia, blood was collected via cardiac puncture, and the spleen was harvested. All animal procedures were conducted in accordance with the “Guidelines for the Care and Use of Laboratory Animals” of the IMB Animal Ethics Committee (License No.: SCXK (dian) K2022-0002).

### 2.5. Mouse Serum Separation

The mouse blood samples were first maintained at 37 °C for one hour and subsequently incubated at 4 °C for two hours. Following this, the blood was placed in a centrifuge and subjected to spinning at 2000 rpm for ten minutes, then increased to 4000 rpm for an additional ten minutes. The serum was carefully transferred to sterile EP tubes in a laminar flow hood.

### 2.6. Mouse Spleen Cell Isolation

Spleen tissue (100 mg) was minced with scissors and transferred to a sterile EP tube. Then, 500 μL of PBS buffer was added, and the tissue was homogenized using a handheld tissue grinder (Tiangen, Beijing, China). An additional 500 μL of PBS was introduced to the mixture, which was then centrifuged at 1500 rpm for five minutes. After discarding the supernatant, the pellet was reconstituted in 100 μL of PBS buffer to form a cell suspension.

### 2.7. Hemagglutination Inhibition (HI) Assay

The receptor-destroying enzyme (RDE) (Seiken, Tokyo, Japan) was combined with mouse serum in a 1:4 volume ratio and placed in a 37 °C water bath for 16 h. Subsequently, the mixture was heated to 56 °C for one hour to deactivate the RDE. Following this, 1/10 volume of freshly prepared chicken red blood cells was added, and the mixture was incubated at 4 °C for 3 h. After centrifugation at 8000 rpm for 10 min, the supernatant was collected under sterile conditions and stored for use.

The serum was serially diluted in two-fold increments (1:10 to 1:2,097,150) with physiological saline in a 96-well microplate (KIRGEN, Brookfield, WI, USA), with 25 μL added to each well. For each mouse, two duplicate wells were set up. Negative control wells (25 μL of physiological saline) and blank control wells (50 μL of physiological saline) were also included. Each well, except for the blank controls, received 25 μL of A/Michigan/45/2015 (H1N1) or A/Victoria/4897/2022 (H1N1) virus containing 8 hemagglutination units (HAU). After leaving the plate at room temperature for one hour, 50 μL of a 1% chicken red blood cell suspension was dispensed into each well. The plate was subsequently incubated at room temperature for thirty minutes. The highest dilution of serum that completely inhibited chicken red blood cell agglutination was considered the corresponding hemagglutination inhibition antibody (HIAb) titer for the sample.

### 2.8. Microneutralization Assay

Receptor-destroying enzyme (RDE) was mixed with mouse serum at a 1:4 volume ratio and incubated in a 37 °C water bath for 16 h. The mixture was then heated at 56 °C for 1 h to inactivate the RDE. Following this, 1/10 volume of freshly prepared chicken red blood cells was added, and the mixture was incubated at 4 °C for 3 h. After centrifugation at 8000 rpm for 10 min, the supernatant was collected under sterile conditions and stored for use.

MDCK cells exhibiting robust growth were plated into a 96-well culture plate at a concentration of 2.5 × 10^5^ cells/mL and left to incubate overnight at 37 °C in a 5% CO_2_ environment. The serum was then serially diluted in virus maintenance medium (DMEM containing 2 μg/mL TPCK-trypsin, 1% BSA, and 1% penicillin-streptomycin) at two-fold dilutions (1:100 to 1:20,971,500) in the plate, with 50 μL per well. Two duplicate wells were set up for each mouse, along with negative control wells (50 μL of physiological saline per well) and blank control wells (100 μL of virus maintenance medium per well). Except for the blank controls, each well received 120 μL of a 200 × TCID_50_ suspension of A/Michigan/45/2015 (H1N1) or A/Victoria/4897/2022 (H1N1) virus. The plate was mixed and incubated at 34 °C for 1 h. The MDCK cells were washed three times with PBS, and 100 μL of the above mixture was added to each well. The plate was then incubated at 34 °C with 5% CO_2_ for 72 h. After incubation, 50 μL of the supernatant from each well was transferred to a 96-well hemagglutination plate, followed by the addition of 50 μL of freshly prepared 1% chicken red blood cell suspension. The plate was incubated at room temperature for 30 min. The highest serum dilution that completely inhibited chicken red blood cell agglutination was considered the corresponding neutralizing antibody (nAb) titer for the sample.

### 2.9. Virus Challenge Experiment

Six groups were formed by randomly assigning mice, with *n* = 10 per group. In the HA group, 30 μg of IAV HA mRNA vaccine was administered intramuscularly in the lower limbs. In the NA group, 30 μg of IAV NA mRNA vaccine was administered intramuscularly in the lower limbs. In the HA+NA group, 30 μg of IAV HA+NA mRNA vaccine was administered intramuscularly in the lower limbs. In the HA/NA group, 15 μg of IAV HA mRNA vaccine was injected in the left lower limb, and 15 μg of IAV NA mRNA vaccine was injected in the right lower limb. The quadrivalent inactivated vaccine group received a single dose of A/Michigan/45/2015 (H1N1) quadrivalent inactivated vaccine in the lower limbs. The LNP group was injected with an equal volume of LNPs. Primary vaccination took place on day 0, and a booster was given on day 14. The body weights of the mice were recorded on day 21 to serve as baseline measurements. Mice were anesthetized, and 5 mice from each group were challenged with 100 LD_50_ of A/Michigan/45/2015 (H1N1) virus by intranasal instillation, while the remaining 5 mice received 100 LD_50_ of A/Victoria/4897/2022 (H1N1) virus. Body weight was monitored daily for 14 days, and a weight loss of more than 20% from the baseline weight was considered as death.

### 2.10. Flow Cytometry for T Cell Subset Analysis

Flow cytometric antibodies were added to the splenocyte suspensions from each group of mice as follows: 4 μL of CD4-APC (1:25) (BioLegend, San Diego, CA, USA), 4 μL of CD3-FITC (1:25) (BioLegend, CA, USA), 2 μL of 7-AAD-PercpCy5 (1:50) (BioLegend, San Diego, CA, USA), 2 μL of CD8α-APC-Cy7 (1:50) (BioLegend, CA, USA), 2 μL of TNF-α-PE (1:50) (BioLegend, San Diego, CA, USA), 2 μL of IL-4-BV605 (1:50) (BioLegend, San Diego, CA, USA), and 2 μL of IFN-γ-PE-Cy7 (1:50) (BioLegend, San Diego, CA, USA). The mixture was incubated at room temperature, protected from light, for 15 min. After mixing, flow cytometry was performed using a NovoCyte D3000 cytometer (Agilent, Santa Clara, CA, USA) to analyze the T cell subsets.

### 2.11. ELISA for Specific IgG Antibody Types and Titers

Both antigens (HA: Influenza A H1N1 A/Michigan/45/2015 (H1N1) Hemagglutinin; NA: Influenza A H1N1 A/Michigan/45/2015 (H1N1) Neuraminidase, SinoBiological, Beijing, China) were diluted to a concentration of 1 μg/mL in 50 mM carbonate buffer (pH 9.5). A total of 100 μL of each antigen was added to each well of a 96-well enzyme-linked immunosorbent assay (ELISA) plate, which was then incubated at 37 °C for 1.5 h for antigen coating. After removing the liquid, the wells were washed three times with 300 μL of PBST (PBS with 0.05% (*v*/*v*) Tween-20) and blotted dry. Next, 100 μL of 2% (*w*/*v*) BSA in 10 mM PBS was added to block the plate, followed by incubation at 37 °C for 1 h. The liquid was removed, and the wells were washed again with PBST as described previously.

Serum samples were diluted starting at 1:100 initial dilution by adding 100 µL to each well, followed by continuous two-fold serial dilutions. The diluent used was PBST containing 0.5% (*w*/*v*) BSA. Each group included two replicate wells. The plate was incubated at 37 °C for 1 h. After removing the liquid, the wells were washed as before. Subsequently, 100 μL of horseradish peroxidase (HRP)-conjugated secondary antibodies (IgG1 or IgG2a, ThermoFisher) at a 1:1000 dilution were added to each well, and the plate was incubated at 37 °C for 1 h. The plate was washed again with PBST, and 100 μL of TMB substrate (SolarBio, Beijing, China) was added to each well, followed by incubation at 37 °C in the dark for 15 min. The reaction was stopped by adding 50 μL of 2 M H_2_SO_4_. Absorbance at 450 nm was measured using an ELISA reader (Bio-Tek Instruments, Winooski, VT, USA). A sample was considered positive if the OD_450_ value was more than 2.1 times that of the LNP group. The highest serum dilution yielding a positive result was recorded as the antibody titer for the corresponding sample.

### 2.12. Immune Dose Optimization

To explore whether the antibody response induced by the multi-site immunization strategy correlates with the immunization dose, a dose optimization experiment was conducted with the HA/NA group. A total of four groups were established by randomly dividing the mice (*n* = 3). On day 0, different doses of mRNA vaccine (0.5 μg, 2.5 μg, 5 μg) and a quadrivalent inactivated vaccine were administered via intramuscular injection. A booster injection was administered on day 14. On day 21, mice were anesthetized, and blood was collected via cardiac puncture. The serum was then isolated, and the HIAb and nAb titers against A/Michigan/45/2015 (H1N1) and A/Victoria/4897/2022 (H1N1) were measured.

### 2.13. Statistical Analysis

Data were analyzed using Prism 9.5.0 (GraphPad, San Diego, CA, USA). For comparisons among multiple groups, one-way ANOVA followed by Tukey’s post-hoc test was performed. For comparisons involving two factors with multiple levels, two-way ANOVA followed by Tukey’s post-hoc test was used. Kaplan–Meier analysis and Log-rank (Mantel–Cox) test were applied to compare survival curves. All results are presented as mean ± standard deviation or geometric mean ± geometric standard deviation. A *p*-value of <0.05 was considered statistically significant. Significance levels are indicated by * *p* < 0.05, ** *p* < 0.01, *** *p* < 0.001, and **** *p* < 0.0001.

## 3. Results

### 3.1. Multi-Site Immunization Strategy Induces High Levels of HIAb in Mice

**Figure 1:**
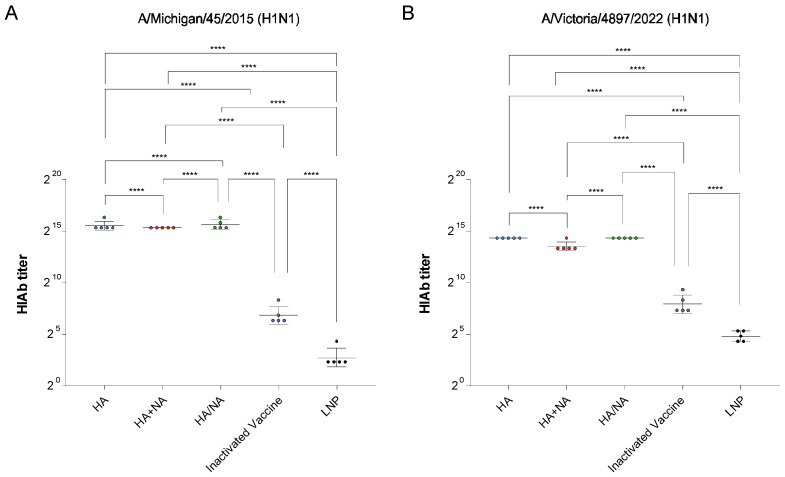
HIAb titers in the sera of mice from the HA, HA+NA, HA/NA, quadrivalent inactivated vaccine, and LNP groups. (**A**) HIAb titers against the matched strain A/Michigan/45/2015 (H1N1). (**B**) HIAb titers against the mismatched strain A/Victoria/4897/2022 (H1N1). An ordinary one-way ANOVA was employed to determine statistical significance using GraphPad Prism version 9.5.0; **** *p* < 0.0001. The results are shown as geometric mean values accompanied by error bars indicating geometric SD.

Since HIAb is a HA antigen-specific antibody, we measured the HIAb titers in the sera of mice from the HA, HA+NA, HA/NA, quadrivalent inactivated vaccine, and LNP groups against the matched strain A/Michigan/45/2015 (H1N1) (Figure 1A) and the mismatched strain A/Victoria/4897/2022 (H1N1) (Figure 1B). Our results showed that when the strain matched, the multi-site immunization strategy induced the highest HIAb titers, followed by the IAV HA mRNA vaccine, and the lowest levels were observed in the IAV HA+NA mRNA vaccine group. In contrast, when the strain was mismatched, the HIAb titers induced by the split-dose strategy were comparable to those of the IAV HA mRNA vaccine, while the IAV HA+NA mRNA vaccine group exhibited the lowest titers.

### 3.2. Multi-Site Immunization Strategy Induces High Levels of nAb in Mice

**Figure 2:**
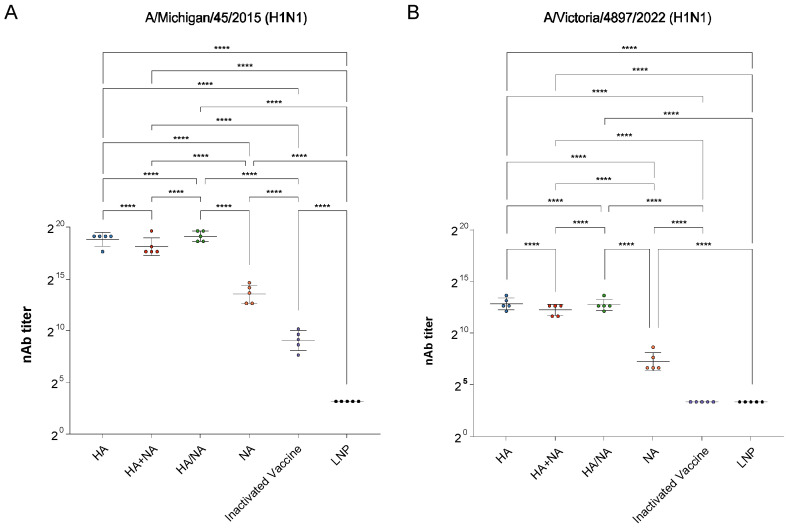
nAb titers in the sera of mice from each group. (**A**) nAb titers against the matched strain A/Michigan/45/2015 (H1N1). (**B**) nAb titers against the mismatched strain A/Victoria/4897/2022 (H1N1). Statistical significance was calculated using an ordinary one-way ANOVA using GraphPad Prism 9.5.0; **** *p* < 0.0001. Data represents geometric mean value and error bars for geometric SD.

To assess the ability of each vaccine to induce nAb in mice, we measured the nAb titers in the sera of mice from each group against the matched strain A/Michigan/45/2015 (H1N1) (Figure 2A) and the mismatched strain A/Victoria/4897/2022 (H1N1) (Figure 2B). We found that, regardless of whether the strain was matched or mismatched, the multi-site immunization strategy induced the highest nAb titers, followed by the IAV HA mRNA vaccine, and the lowest levels were observed in the IAV HA+NA mRNA vaccine group. Although the nAb titers induced by the IAV NA mRNA vaccine were lower than those of the aforementioned three groups, they were still higher than those induced by the quadrivalent inactivated vaccine, which is consistent with our previous findings.

### 3.3. All mRNA Vaccines Can Provide the Same Protection to Mice in the Virus Challenge as the Quadrivalent Inactivated Vaccine

**Figure 3:**
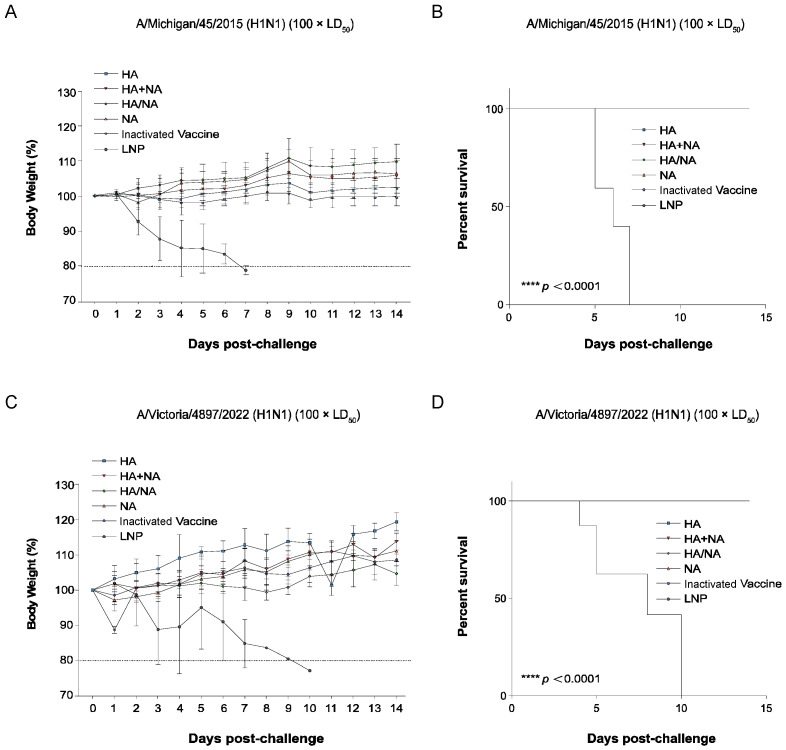
Body weight and survival of mice in the virus challenge experiment. (**A**) Statistical analysis of body weight changes in mice after challenge with A/Michigan/45/2015 (H1N1). The horizon dash line is 80% of the initial body weight, values below this threshold are considered as death. Data represents the mean value and error bars for standard deviation (SD). (**B**) Survival curve of mice in the A/Michigan/45/2015 (H1N1) virus challenge experiment. Kaplan–Meier analysis using GraphPad Prism 9.5.0 indicated significant differences in survival curves between HA, HA+NA, HA/NA, NA Inactivated Vaccine group, and the LNP group (**** *p* < 0.0001). (**C**) Statistical analysis of body weight changes in mice after challenge with A/Victoria/4897/2022 (H1N1). Data represents the mean value and error bars for SD. The horizon dash line is 80% of the initial body weight, values below this threshold are considered as death. (**D**) Survival curve of mice in the A/Victoria/4897/2022 (H1N1) virus challenge experiment. Kaplan-Meier analysis using GraphPad Prism 9.5.0 indicated significant differences in survival curves between HA, HA+NA, HA/NA, NA Inactivated Vaccine group, and the LNP group (**** *p* < 0.0001).

To evaluate the protective effects of each vaccine in the virus challenge experiment, we measured the body weight of the mice on day seven after the booster immunization, which was used as the baseline. Mice were then challenged with a nasal drop of 100LD_50_ of the matched strain A/Michigan/45/2015 (H1N1) and the mismatched strain A/Victoria/4897/2022 (H1N1). Body weight was monitored for 14 days, and a weight loss of less than 80% of the initial body weight was considered as death (Figure 3A,C). Survival curves were plotted (Figure 3B,D). The results showed that all mice in the LNP group died within 14 days post-challenge, whereas all other groups survived.

### 3.4. Multi-Site Immunization Strategy Stimulates the Production of Different T Cell Types in Mice

**Figure 4:**
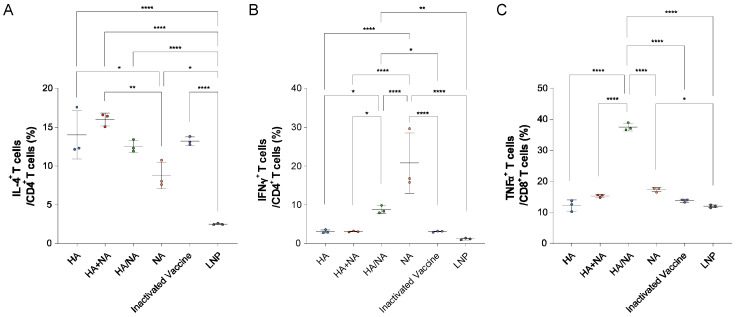
Flow cytometric identification of T cell subtypes induced in mice. (**A**) Levels of IL4^+^/CD4^+^ T cells induced in mice from each group. (**B**) Levels of IFNγ^+^/CD4^+^ T cells induced in mice from each group. (**C**) Levels of TNFα^+^/CD8^+^ T cells induced in mice from each group. Statistical significance was calculated using an ordinary one-way ANOVA using GraphPad Prism 9.5.0; * *p* < 0.05; ** *p* < 0.01; **** *p* < 0.0001. Data represents the mean value and error bars for standard deviation (SD).

On day seven after the booster immunization, we used flow cytometry to identify T cell subsets in mice from each group, including IL4^+^/CD4^+^ T cells (Figure 4A), IFNγ^+^/CD4^+^ T cells (Figure 4B), and TNFα^+^/CD8^+^ T cells (Figure 4C). We found that the HA antigen primarily induced the production of IL4^+^/CD4^+^ T cells, whereas the NA antigen primarily induced IFNγ^+^/CD4^+^ T cells. In theory, both the IAV HA+NA mRNA vaccine and the multi-site immunization strategy should induce both types of T cells. However, only the multi-site immunization strategy achieved this effect, and it also induced the production of TNFα^+^/CD8^+^ T cells. These results suggest that the multi-site immunization strategy can generate cytotoxic T cells and elicit a balanced Th1/Th2 immune response.

### 3.5. Multi-Site Immunization Strategy Induces Both IgG1 and IgG2a Antibodies in Mice

**Figure 5:**
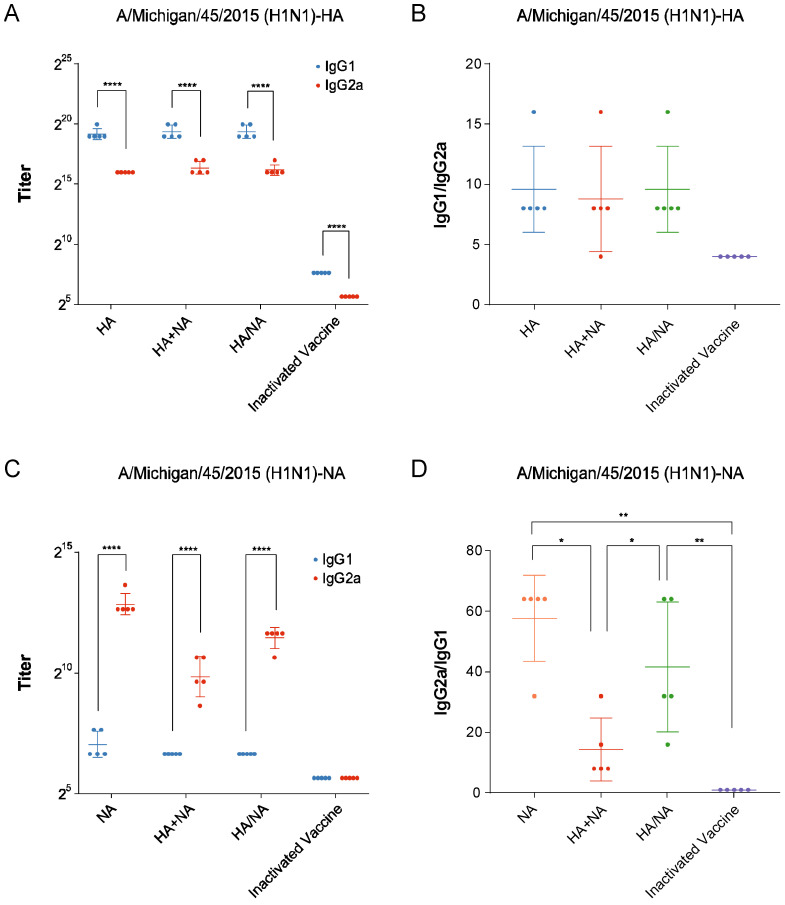
Measurement of specific anti-HA and anti-NA antibodies and their titers in mouse serum. (**A**) Titers of IgG1 and IgG2a antibodies specific to HA in the serum of mice from each group. Statistical significance was calculated using a Two-way ANOVA using GraphPad Prism 9.5.0; **** *p* < 0.0001. Data represents geometric mean value and error bars for geometric SD. (**B**) IgG1/IgG2a ratio for HA-specific antibodies in the serum of mice from each group. Statistical significance was calculated using an ordinary one-way ANOVA using GraphPad Prism 9.5.0. Data represents the mean value and error bars for standard deviation (SD). (**C**) Titers of IgG1 and IgG2a antibodies specific to NA in the serum of mice from each group. Statistical significance was calculated using a Two-way ANOVA using GraphPad Prism 9.5.0; **** *p* < 0.0001. Data represents geometric mean value and error bars for geometric SD. (**D**) IgG2a/IgG1 ratio for NA-specific antibodies in the serum of mice from each group. Statistical significance was calculated using an ordinary one-way ANOVA using GraphPad Prism 9.5.0; * *p* < 0.05; ** *p* < 0.01. Data represents the mean value and error bars for standard deviation (SD).

To further assess the balance of the Th1/Th2, we performed ELISA to determine the types and titers of specific anti-HA and anti-NA antibodies in the serum of mice from each group. We found that the HA antigen primarily induced the production of IgG1 antibodies (Figure 5A,B), while the NA antigen primarily induced the production of IgG2a antibodies (Figure 5C,D). In theory, both the IAV HA+NA mRNA vaccine and the multi-site immunization strategy should induce the production of both antibody types. However, only the multi-site immunization strategy achieved this effect, further supporting the conclusion that this strategy induces a balanced Th1/Th2 immune response.

### 3.6. Dose Optimization of the Multi-Site Immunization Strategy

**Figure 6:**
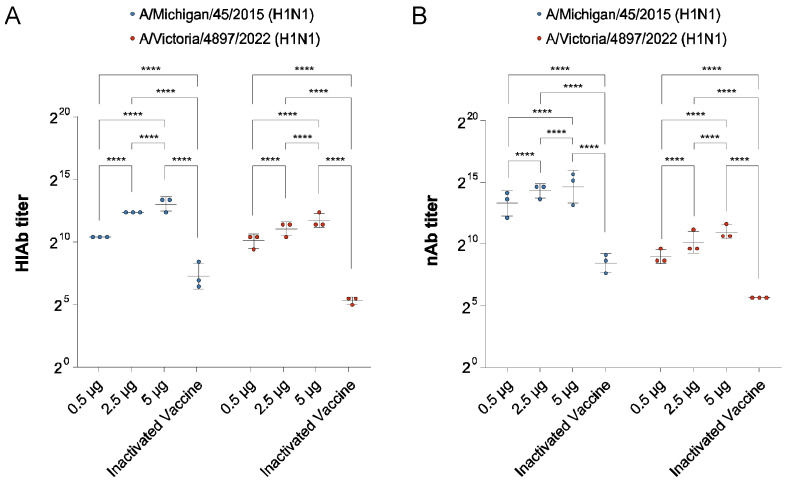
Antibody levels in mouse serum after split-dose immunization with different doses and quadrivalent inactivated vaccine. (**A**) HIAb titers in mouse serum against A/Michigan/45/2015 (H1N1) and A/Victoria/4897/2022 (H1N1). (**B**) nAb titers in mouse serum against A/Michigan/45/2015 (H1N1) and A/Victoria/4897/2022 (H1N1). Statistical significance was calculated using a Two-way ANOVA using GraphPad Prism 9.5.0; **** *p* < 0.0001. Data represents geometric mean value and error bars for geometric SD.

Our previous work demonstrated that the multi-site immunization strategy could induce high levels of HIAb and nAb in mice, as well as cytotoxic T cells and a balanced Th1/Th2 immune response. This result was based on an immunization dose of 30 μg. However, in practical applications, such a high immunization dose may not be necessary. Therefore, we set up three lower immunization doses (0.5 μg, 2.5 μg, and 5 μg) for the immunization process, measuring the HIAb and nAb titers against A/Michigan/45/2015 (H1N1) and A/Victoria/4897/2022 (H1N1) in the serum, and compared them with the quadrivalent inactivated vaccine. The experimental results showed that the HIAb titers against both strains increased with the immunization dose, and all dose groups were significantly higher than the four-valent inactivated vaccine group (Figure 6A). Similarly, the nAb titers against both strains also increased with the immunization dose, and all dose groups were significantly higher than the quadrivalent inactivated vaccine group (Figure 6B). These results indicate that even at an immunization dose as low as 0.5 μg, the multi-site immunization strategy still induced higher HIAb and nAb titers than the quadrivalent inactivated vaccine.

## 4. Discussion

Currently available influenza vaccines primarily target the hemagglutinin (HA) of the influenza virus. Nevertheless, HA is prone to antigenic drift, leading to mismatches between the vaccine strain and circulating strains, posing a significant challenge for the production and application of current influenza vaccines. This underscores the urgent need for a broadly protective influenza vaccine that can target various influenza virus strains. The development of broad-spectrum influenza vaccines has focused on several conserved influenza proteins, such as nucleoprotein (NP), matrix proteins M2 and M1 stalk, and polymerase basic protein 1 (PB1) [22]. However, neuraminidase (NA), another major antigen of the influenza virus, has often been overlooked. As early as shortly after the 1968 H3N2 pandemic, researchers discovered that NA antibodies against H2N2 could help protect 300 adults from H3N2 infection [23,24]. Since then, it has been known that NA antibodies could provide broad protection. Yet, it was not until the past decade that the importance of NA in influenza vaccine development gained more attention. Recent studies have identified several broadly protective human antibodies against NA, suggesting that NA may indeed be a key target for the formulation of a widely protective vaccine against influenza [25,26].

In addition, most of the currently available influenza vaccines are inactivated vaccines (IIV). After administration of IIV, antigens are primarily presented on the surface of antigen-presenting cells (APCs) via the MHCII pathway, primarily stimulating CD4^+^ T cells [27]. However, IIV predominantly induces a Th2-type immune response, leading to the production of specific antibodies against the vaccinated influenza strain, thereby providing immune protection [28,29]. Despite this, the average efficacy of influenza vaccines over the past decade has been only 42%, with even lower protection rates for young children and the elderly [30]. For influenza vaccines, serological protection, seroconversion rates, and geometric mean titers are the key criteria for market approval. Currently, the focus of vaccine evaluation is on the Th2-type immune response rather than Th1-type immunity, yet Th1 responses also play a crucial role in defending against influenza viruses [31]. Therefore, influenza vaccines capable of inducing a balanced Th1/Th2 immune response across all age groups could significantly reduce morbidity and mortality. Live attenuated influenza vaccines (LAIV), administered via the intranasal route, infect upper respiratory tract cells and process antigens more effectively through the MHCI pathway. They induce both mucosal and cellular immunity, activate Th1-type immune responses, and promote a balanced Th1/Th2 immune response [32,33]. This makes LAIV an ideal vaccine type. However, LAIV faces safety issues, as it is not suitable for young children or immunocompromised individuals, and developing LAIV for avian influenza is particularly challenging due to its tendency to affect the lower respiratory tract in humans more than the upper respiratory tract [34]. Thus, advancing new influenza vaccines that can stimulate stronger and more harmonized Th1/Th2 immune responses will likely be a key focus in the future.

Our previous studies have shown that the IAV NA mRNA vaccine can induce a Th1-type immune response in mice. Therefore, in this study, we aimed to combine the advantages of both HA and NA to design an influenza vaccine that induces a more balanced Th1/Th2 immune response and provides broad protective efficacy. We designed a dual-antigen mRNA vaccine capable of expressing both HA and NA antigens, employing two different immunization strategies. One approach involved linking HA and NA antigens using P2A to create the IAV HA+NA mRNA vaccine, which was injected into the right hind limb of mice. The other strategy was a split-dose immunization, where IAV HA mRNA vaccine was injected into the left hind limb, and IAV NA mRNA vaccine was injected into the right hind limb of the mice. We compared the immune responses induced by these two dual-antigen immunization methods with those induced by right hind limb injections of IAV HA mRNA vaccine, IAV NA mRNA vaccine, and the quadrivalent inactivated vaccine. The results showed that the multi-site immunization strategy induced higher levels of HIAb and nAb in mice and was more effective in inducing cytotoxic T cells and a balanced Th1/Th2 immune response.

In this regard, we hypothesize that the IAV HA+NA mRNA vaccine simulates the antigenic competition between HA and NA within the virus during antigen expression in vivo. These antigens are absorbed and presented by the same antigen-presenting cells (APCs), with viral HA being superior to NA in eliciting both T cell and B cell responses [35]. Studies have shown that this intra-viral antigen competition can be improved by separating the HA and NA proteins, which is why, with the multi-site immunization strategy, the suppression of the immune response to NA is reduced, resulting in higher levels of antibodies and the induction of a balanced Th1/Th2 response along with cytotoxic T cells [17]. These results suggest that the multi-site immunization strategy can generate cytotoxic T cells and a balanced Th1/Th2 immune response. However, in the flow cytometry analysis of T cell types, while the split-dose strategy induced significantly higher levels of IFNγ^+^/CD4^+^ T cells compared to the IAV HA mRNA and IAV HA+NA mRNA vaccines, the level remained lower than that induced by the IAV NA mRNA vaccine. This indicates that, although the split-dose strategy reduces the suppression of the NA immune response by HA to some extent, it does not eliminate it completely. This may be related to the immune dose ratio between the IAV HA mRNA vaccine and IAV NA mRNA vaccine. Future studies could explore a series of different immune dose ratios. Additionally, it is interesting that IAV HA mRNA vaccine and IAV NA mRNA vaccine on their own did not induce strong CD8 responses, but the split-dose immunization elicited significantly higher level of TNF-α^+^/CD8^+^ T cells than the other groups. Our hypothesis regarding this point is as follows. IFN-γ secreted by Th1 cells can enhance the activity of CD8⁺ T cells, promoting their TNFα secretion and cytotoxic functions. In contrast, IL-4 and IL-10 produced by Th2 cells suppress Th1 cell differentiation and function, thereby indirectly inhibiting CD8⁺ T cell activation and TNFα secretion. Under Th2-skewed immune responses, the effector functions of CD8⁺ T cells may be diminished. However, our findings demonstrated that during split-dose immunization, the suppression of Th1 immunity by Th2 responses was reduced, thus, resulting TNFα⁺/CD8⁺ response elicited by the IAV NA mRNA vaccine, which primarily induced a Th1-type immune response. We hypothesize that the simultaneous presence of both Th1- and Th2-type immune responses leads to an increased diversity and quantity of inflammatory cytokines. Specifically, the combined presence of IL-2, IL-12, and IL-4 may provide stronger proliferative and effector signals for CD8⁺ T cells, thereby enhancing TNFα production. Moreover, IL-4 may upregulate the expression of MHC I molecules on antigen-presenting cells, further improving the ability of CD8⁺ T cells to recognize antigens. In the split-dose immunization dose exploration experiment, although the HIAb and nAb titers induced by a 0.5 μg dose were higher than those induced by the quadrivalent inactivated vaccine, further flow cytometry analysis is needed to confirm whether this dose can induce cytotoxic T cells and a balanced Th1/Th2 immune response in mice.

In terms of inducing broad-spectrum protection, we designed the dual-antigen mRNA vaccine targeting both HA and NA based solely on the A/Michigan/45/2015 (H1N1) strain. Future studies could design vaccines targeting multiple circulating strains to potentially offer broader protection. Additionally, because only a limited variety of strains were examined, this study only evaluated the cross-reactive antibody levels induced in mice against the A/Victoria/4897/2022 (H1N1) strain and the protection conferred in the virus challenge experiment.

## 5. Conclusions

This research involved the creation of a dual-antigen mRNA vaccine targeting IAV and the evaluation of two distinct immunization approaches. One approach involved linking the HA and NA antigens using P2A to create the IAV HA+NA mRNA vaccine, which was injected into the right hind limb of mice. The other strategy employed a split-dose immunization, where IAV HA mRNA vaccine was injected into the left hind limb and IAV NA mRNA vaccine was injected into the right hind limb. We compared the immunization efficacy of these two dual-antigen methods with those of right hind limb injections of IAV HA mRNA vaccine, IAV NA mRNA vaccine, and the quadrivalent inactivated vaccine. The results showed that the multi-site immunization strategy induced high levels of HIAb and nAb in mice against both matched and mismatched strains, provided protection in the virus challenge experiments, and stimulated the production of cytotoxic T cells as well as a balanced Th1/Th2 immune response. We then explored the optimal immunization dose for this method, and found that the HIAb and nAb titers in mouse serum increased with the immune dose. Even at a low dose of 0.5 μg, the HIAb and nAb levels were significantly higher than those induced by the quadrivalent inactivated vaccine, indicating that our dual-antigen mRNA vaccine, when administered using the split-dose strategy at a low dose, exhibits superior immunogenicity compared to the existing quadrivalent inactivated vaccine.

## Data Availability

The data used to support the findings of this study are included within the article.
